# Evaluating Image Quality and Radiation Dose in Low-Dose Thoraco-Abdominal CT Angiography with a Tin Filter for Patients with Aortic Disease

**DOI:** 10.3390/jcm13040996

**Published:** 2024-02-08

**Authors:** Chang Hoon Oh, Soo Buem Cho, Hyeyoung Kwon

**Affiliations:** 1 Department of Radiology, Ewha Womans Mokdong Hospital, College of Medicine, Ewha Womans University, Seoul 07985, Republic of Korea; 01352@ewha.ac.kr; 2 Department of Radiology, Ewha Womans Seoul Hospital, College of Medicine, Ewha Womans University, Seoul 07804, Republic of Korea; 3 Department of Radiology, Chungnam University Hospital, School of Medicine, Chungnam University, Daejeon 35015, Republic of Korea; hyeyoungkwon@cnuh.ac.kr

**Keywords:** tin filter, thoraco-abdominal CT angiography, radiation exposure, image quality, diagnostic imaging

## Abstract

**Background:** We aimed to compared radiation exposure and image quality between tin-filter-based and standard dose thoraco-abdominal computed tomography angiography (TACTA) protocols, aiming to address a gap in the existing literature. **Methods:** In this retrospective study, ninety consecutive patients undergoing TACTA were included. Of these, 45 followed a routine standard-dose protocol (ST100kV), and 45 underwent a low-dose protocol with a tin filter (TF100kV). Radiation metrics were compared. The signal-to-noise ratio (SNR), contrast-to-noise ratio (CNR), and figure of merit (FOM) were calculated for the thoracic and abdominal aorta and right common iliac artery. Two independent readers assessed the image noise, image contrast, sharpness, and subjective image quality. **Results:** The mean dose for the TF100kV group was significantly lower (DLP 128.25 ± 18.18 mGy*cm vs. 662.75 ± 181.29, *p* < 0.001; CTDIvol 1.83 ± 0.25 mGy vs. 9.28 ± 2.17, *p* = 0.001), with an effective dose close to 2.3 mSv (2.31 ± 0.33 mSv; *p* < 0.001). The TF100kV group demonstrated greater dose efficiency (FOM, thoracic aorta: 36.70 ± 22.77 vs. 13.96 ± 13.18 mSv^−1^, *p* < 0.001) compared to the ST100kV group. **Conclusions:** Dedicated low-dose TACTA using a tin filter can significantly reduce the radiation dose while maintaining sufficient diagnostic image quality.

## 1. Introduction

Computed tomography (CT) angiography of the thoraco-abdominal aorta is crucial for diagnosing and managing acute, life-threatening aortic conditions, such as penetrating atherosclerotic ulcers (PAU), aortic dissections (AD), and acute intramural hematomas (IMH), among others. These conditions, often presenting with similar symptoms, can lead to severe complications [1]. A comprehensive evaluation of these disorders necessitates CT scans from the thoracic to the abdominal aorta, often including pre-contrast imaging. Given the high levels of radiation exposure associated with multiple scans, minimizing the radiation dose remains a critical concern.

Recent years have seen significant technological advancements in CT systems for clinical use. Iterative reconstruction algorithms have now supplanted filtered back projection in CT image reconstruction [2,3]. In fact, all modern CT systems, irrespective of the manufacturer, incorporate some form of pre-filtration. This pre-filtration strengthens the beam and absorbs low-energy photons, which minimally contribute to the final image but increase patient dose [4]. A notable innovation is the standard inclusion of an additional built-in tin filter (TF) on the X-ray tube in all new CT systems [5]. This TF, especially at a fixed tube voltage of 100 kVp, effectively removes the majority of lower-energy photons by utilizing a 0.6 mm thick layer, resulting in a mean photon energy of 78.7 keV. This energy level is significantly higher compared to the mean energy of a standard 100 kVp examination, which is 66.4 keV [6], indicating a substantial reduction in radiation dose.

While previous studies have primarily focused on ultralow dose chest CT, coronary artery calcium scoring CT, and non-enhanced studies such as those for urinary calculi and osteolytic lesions in cases of multiple myeloma [4,7,8,9], there have been reports of significant radiation dose reduction without notable differences in image quality in abdominal–pelvic CT (APCT) for oncological follow-up and chest–abdominal–pelvic (CAP) CT in patients with colorectal cancer using contrast media [10,11]. However, the efficacy of TF-based thoraco-abdominal aorta CT angiography (TACTA) compared to standard protocol TACTA has not been comprehensively examined. This study, therefore, investigates the radiation exposure and image quality of TF-based spectral-shaping TACTA in comparison to standard protocols.

## 2. Materials and Methods

This study received approval from our institutional review board, which waived the requirement for written informed consent due to its retrospective nature (SEUMC 2023-11-003-001).

### 2.1. Patient Population

A retrospective, technical efficacy study was conducted on patients referred for CT angiography of the thoraco-abdominal aorta. Our PACS database was reviewed from December 2021 to February 2022. Referral indications included suspicion of acute aortic syndrome, such as PAU, IMH, and AD, preoperative work-up in aortic aneurysm cases, postoperative aortic aneurysm evaluation, and imaging of the renal and gastrointestinal arteries. General exclusion criteria were an allergy to contrast media, mental incompetence, known arrhythmias or other heart disorders, impaired renal function (estimated glomerular filtration rate <60 mL/min), age below 18 years, and pregnancy or lactation.

Patient characteristics, including sex, age, height, weight, and body mass index (BMI), were collected from electronic medical records. The study included a total of 90 consecutively registered BMI-matched patients. Two groups were formed, each comprising 45 patients: one group underwent TACTA using a routine standard-dose protocol (ST100kV), and the other underwent a low-dose protocol with a tin filter (TF100kV). Advanced modeled iterative reconstruction with a strength level of 3 was applied in both groups. To estimate radiation doses, dose parameters for each patient and acquisition protocol were recorded. Volume CT dose index (CTDIvol), dose-length product (DLP), and effective tube current (mAs) were extracted from the dose report and DICOM data. The effective dose (ED) of CT was calculated using a method derived from the European Working Group, employing the adult abdominopelvic CT weighting factor (k = 0.018 mSv mGy^−1^ cm^−1^) [12].

### 2.2. CT Examinations

All patients underwent a single examination using a single-source CT system (Siemens SOMATOM Force, Siemens Healthineers, Erlangen, Germany) at a fixed tube potential of 100 kVp, utilizing tube current modulation software (CareDose4D; Siemens Healthcare). Each patient received a three-phase CT scan, comprising unenhanced, arterial, and delayed phases. The unenhanced scans were performed first. For the arterial phase, a bolus-triggering technique was employed, initiating the scan 12 s after reaching a trigger threshold of 100 HU at the abdominal aorta. This phase used 120 mL of contrast medium (Omnihexol 350 mg I/mL; 600 mg I/kg based on a 70 kg standard) injected intravenously at a rate of 4 mL/s using an automatic power injector. The delayed phases were obtained 180 s after the administration of the contrast medium. Both the TF100kV and ST100kV protocols covered the entire CAP area, ranging from the upper level of the thyroid to the great trochanter. By default, the images from both the TF100kV and ST100kV protocols were reconstructed with a slice thickness of 2.0 mm and without any interslice gap.

### 2.3. Qualitative Analysis

All 90 thoraco-abdominal aorta CT examinations were evaluated by two radiologists, each with significant experience in abdominal CT interpretation (one with 14 years and the other with 7 years of experience), and both were blinded for peer review. All reviews were conducted on our clinical Picture Archiving and Communication System (PACS, INFINITT Healthcare, Seoul, Republic of Korea). Subjective assessments of image noise, overall image quality, image contrast, and sharpness were evaluated (Table 1). Image contrast was assessed by examining the differentiation between the superior mesenteric artery (SMA) and mesentery, specifically focusing on the depiction of the segmental branch level of the SMA, using a 5-point scale. Image sharpness was evaluated by examining the clarity of the liver contour, also using a 5-point scale. Similarly, the overall image quality was rated using a 5-point scale.

### 2.4. Quantitative Analysis

A radiologist with seven years of experience in abdominal radiology (blinded for peer review) conducted a quantitative analysis to determine the noise, contrast-to-noise ratio (CNR), and signal-to-noise ratio (SNR). To assess CNR and SNR, one region of interest (ROI) was drawn on the vessel (ROIvessel) and a second ROI within the muscle at the same slice location (ROImuscle) on the arterial phase image. These ROIs, with an area of 1–3 cm^2^, were placed manually at three different locations: the thoracic aorta (aortic arch level), abdominal aorta (celiac os level), and right common iliac artery (CIA). The mean standard deviation (SD) of the three vessel ROI measurements represents the image noise.

SNRvessel = ROIvessel/standard deviation (ROIvessel)

CNRvessel = (ROIvessel − ROImuscle)/standard deviation (ROIvessel)

A figure of merit (FOM) was calculated as CNR2/effective dose [13] to compare dose efficiency between the two acquisition protocols.

**Figure 1:**
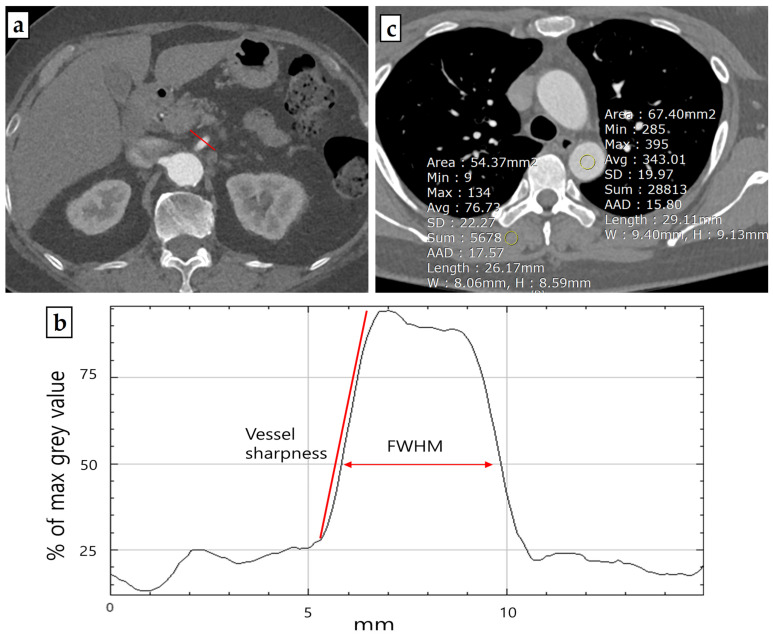
Representative example of a thoraco-abdominal aorta computed tomography angiography (**a**) (in this case arterial phase scan) used for quantitative image quality analysis. Based on a line profile perpendicular (red line) to the proximal SMA, vessel sharpness and FWHM values were derived (**b**). Representative example of ROI placement on source images for signal-to-noise ratio (SNR) and contrast-to-noise ratio (CNR) measurements (**c**).

For the estimation of vessel sharpness (VS) [14], a line profile perpendicular to the proximal SMA within 2 cm from the SMA os was generated using FIJI’s [15] “Line Profile” function (Figure 1). The slope of the regression line for the anterior vessel border (sloperise) and the posterior vessel border (slopefall) was calculated with Excel’s built-in “slope function”. The mean of the sloperise and the absolute value of the slopefall were calculated to report quantitative numbers for VS. The proximal SMA was chosen for its good delineation due to the surrounding mesenteric fat, making it suitable for evaluating vessel sharpness. All patients were measured with a window width of 400 HU and a window level of 60 HU in the arterial phase.

Vessel sharpness = mean (sloperise + abs(slopefall))

The Full-Width at Half Maximum (FWHM) edge criterion is a quantitative metric that provides a reliable and robust estimation of a vessel lumen’s edge by defining the boundary at a 50% intensity level between the maximum (lumen) and minimum (tissue). This criterion allows for accurate detection of the vessel lumen and derivation of vessel parameters such as diameter or cross-sectional area [16,17]. To generate FWHM values, the line profile from the proximal SMA computed with FIJI’s “Line Profile” function was used. The length (in millimeters) at the half maximum of the line profile curve was measured to reveal the FWHM value. 

### 2.5. Statistical Analysis

Continuous variables are presented as mean ± standard deviation and were compared using Student’s *t*-test. Categorical variables were analyzed using the chi-squared test or Fisher’s exact test where appropriate. *p* < 0.05 was considered statistically significant. Dose parameters, overall image quality, and quantitative measurements, including CNR, SNR, FOM, VS, and FWHM, were compared using an independent *t*-test for parametric analysis.

Inter-reader agreement for the readers’ scores was assessed using a linear weighted kappa (κ) statistic for qualitative analysis of image quality. The agreement was categorized as follows: 0–0.2 (poor), 0.21–0.40 (fair), 0.41–0.60 (moderate), 0.61–0.80 (good), and 0.81–1.00 (excellent). All statistical analyses were performed using commercially available software (IBM SPSS Statistics for Windows, v. 26.0; IBM, Armonk, NY, USA; or MedCalc, v. 19.2.1; MedCalc, Marikerke, Belgium).

## 3. Results

**Figure 2:**
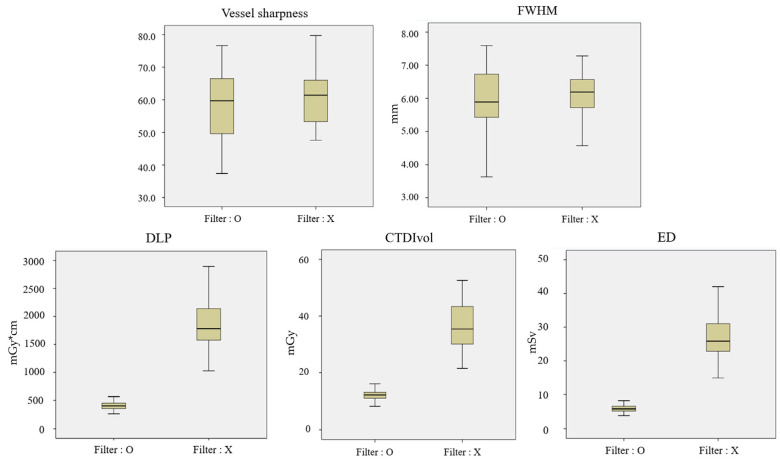
Box and whisker diagrams of DLP, CTDIvol, ED, VS, FWMH for the tin-filtered 100 kV (TF100kV) and standard 100 kV (ST100kV) protocols.

The demographic characteristics of the study population and radiation dose estimates are presented in Table 2. There were no significant differences between the two cohorts in terms of age (*p* = 0.583), sex (*p* = 1.000), or BMI (*p* = 0.857). However, the CT findings for TACTA varied significantly between the groups (*p* = 0.001). Notably, there were various findings, such as aortic aneurysms, acute aortic syndromes, and cases requiring postoperative follow-up after aortic surgery, which necessitated the scans. In the TF100kV group, two cases following EVAR procedures exhibited endoleaks, and two cases necessitated emergency TEVAR due to thoracic aorta dissection (Figure 2).

All radiation dose estimate factors exhibited significant differences between the two groups. The mean CTDIvol was 1.83 ± 0.25 mGy for the TF100kV protocol, markedly lower than the 9.28 ± 2.17 mGy for the ST100kV protocol, a difference that was statistically significant (*p* = 0.001). Similarly, the mean DLP for the TF100kV protocol was significantly lower than that of the ST100kV protocol (128.25 ± 18.18 mGy cm vs. 662.75 ± 181.29 mGy cm, *p* < 0.001). The mean effective dose followed this trend, with 2.31 ± 0.33 mSv for the TF100kV protocol compared to 11.93 ± 3.26 mSv for the ST100kV protocol (*p* < 0.001), representing an 80.6% reduction in dose when using the TF100kV protocol vs. the ST100kV protocol (Table 2 and Figure 2). Contrary to these factors, the mAs for the TF100kV protocol was significantly higher, at 428.71 ± 61.81, compared to 376.20 ± 25.73 for the ST100kV protocol (*p* < 0.001).

In the qualitative analysis, there was no significant difference in image noise, overall image quality, or vessel sharpness between the groups, regardless of the presence or absence of a filter (Table 3). The mean scores for image noise and contrast and sharpness in the ST100kV group were significantly higher than those in the TF100kV group. Specifically, for image noise, the scores were 4.34 ± 0.58 vs. 3.86 ± 0.89 (*p* = 0.026), and for contrast and sharpness, 4.37 ± 0.57 vs. 3.93 ± 0.74 (*p* = 0.008), respectively. The inter-reader agreement between the two reviewers was moderate to good, or even excellent, for image noise (κ: 0.535) and image sharpness (κ: 0.785). The score of subjective image quality in the ST100kV group was also higher than that in the TF100kV group, but statistically no significance was found. The mean scores were 4.04 ± 0.77 vs. 4.22 ± 0.62 (*p* = 0.232), respectively. The inter-reader agreement between the two reviewers was good for image noise (κ: 0.693).

In the quantitative image analysis, the SNRs of the thoracic and abdominal aorta and the CIA were significantly higher for the ST100kV protocol compared to those for the TF100kV protocol. Specifically, the thoracic aorta showed an SNR of 23.16 ± 8.52 vs. 18.27 ± 3.89 (*p* = 0.001), the abdominal aorta had 18.13 ± 5.39 vs. 15.95 ± 3.29 (*p* = 0.023), and the CIA had 22.25 ± 8.19 vs. 17.62 ± 4.93 (*p* = 0.002), respectively. The CNRs for the thoracic and abdominal aorta and CIA were also significantly higher for the ST100kV protocol than those for the TF100kV protocol: thoracic aorta at 19.72 ± 7.25 vs. 15.22 ± 3.68 (*p* < 0.001), abdominal aorta at 15.52 ± 4.88 vs. 13.25 ± 4.88 (*p* = 0.011), and CIA at 18.76 ± 7.02 vs. 14.37 ± 4.47 (*p* = 0.001), respectively. However, the mean FOMs for the thoracic and abdominal aorta and CIA were significantly higher for the TF100kV protocol than those for the ST100kV protocol thoracic aorta at 36.70 ± 22.77 mSv^−1^ vs. 13.96 ± 13.18 mSv^−1^ (*p* < 0.001), abdominal aorta at 27.89 ± 15.65 mSv^−1^ vs. 7.94 ± 4.82 mSv^−1^ (*p* < 0.001), and CIA at 34.59 ± 27.02 mSv^−1^ vs. 12.56 ± 11.55 mSv^−1^ (*p* < 0.001), respectively. VS and FWHM at the proximal SMA were significantly higher for the ST100kV protocol (VS: 64.23 ± 8.12 vs. 58.50 ± 10.06, *p* = 0.004; FWHM: 6.59 ± 0.89 vs. 5.89 ± 1.10, *p* = 0.001, respectively) (Table 4).

## 4. Discussion

The findings of our study indicate that implementing tin filtration in TACTA for aortic or arterial disease evaluation can result in an 80.6% reduction in radiation dose compared to the ST100kV. This reduction aligns with the 32–89% range reported in previous studies utilizing a TF, underscoring the significant impact of a TF in lowering radiation doses [4,7,8,9,10,11]. In terms of quantitative analysis, the SNR, CNR, VS, and FWHM were significantly higher in the ST100kV group than those in the TF100kV group (*p* < 0.001). However, the FOM was notably higher in the TF100kV group for both the thoracic and abdominal aorta as well as the right CIA, indicating greater dose efficiency in the TF100kV group compared to that in the ST100kV group. Additionally, subjective image quality factors of qualitative analysis showed no significant differences between the TF100kV and ST100kV groups in our study. This suggests that, despite a general decline in both quantitative and qualitative factors, there is no substantial shortfall in achieving better dose efficiency and in maintaining a distinct standard in interpretation.

In light of recent advancements, our study aligns with earlier research that utilized TF in CT scans and employed contrast agents for image enhancement. While Leyendecker et al. [10] reported no significant differences in CNR or background noise between the TF100kV and standard contrast-enhanced APCT protocols, Kimura et al. [11] observed a lower CNR and increased background noise with the TF100kV protocol. These variances could be due to differences in CT scanner technologies, protocol standardization, and the use of a third-generation dual-source CT system in the studies by Leyendecker et al. Notably, our study achieved a radiation dose reduction of 80.6%, comparable to the reductions of 81.0% and 89.2% reported in previous research, without significant compromise in qualitative image analysis. Contrasting with earlier studies that predominantly focused on cancer patients and the assessment of solid organ or bone tumors, our study broadens the scope to include arterial and aortic diseases. Remarkably, despite incorporating a TF, we observed no qualitative differences in image quality, underscoring the effectiveness of a TF in these applications. Although some studies have been conducted using a tin filter, the advantage of significantly reducing the radiation dose without compromising image quality to a readable level suggests that it could be beneficial for radiation-sensitive organs or women of childbearing age. However, further research is needed in this area.

**Figure 3:**
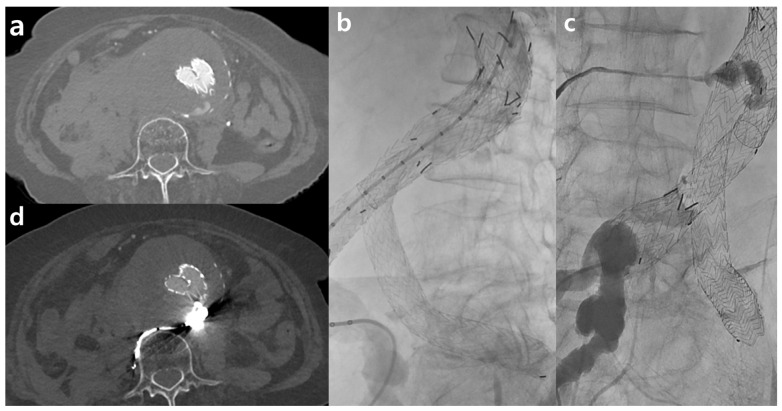
Examples of third-generation dual-source computed tomography clinical applications include Sn100 spectral shaping in a patient with a type II endoleak following endovascular aneurysm repair (EVAR). After EVAR for an abdominal aorta aneurysm, there appeared to be contrast media leakage inside the aneurysm sac, suggesting an endoleak (**a**). The patient was suspected to have an accompanying aneurysmal sac rupture and thus underwent embolization. During aortography, contrast leakage was observed in the delayed phase (**b**). This seemed to originate from the right iliolumbar artery, and embolization was performed using Onyx after selection (**c**). A follow-up was conducted two days later using the Sn100kV protocol, where the radiopaque Onyx was visible, and no further contrast media leakage was observed (**d**).

The pivotal findings of our retrospective study highlight that using Sn 100 kVP is comparable to standard protocols in diagnosing aortic or arterial diseases and preoperative planning. This comparability considers objective parameters, dose exposure, and clinical implications. In our cohort, 40% of patients undergoing either endovascular or surgical repair for acute aortic syndrome encountered no challenges in postoperative assessments, such as detecting endoleaks, even when TACTA with a TF was used. Prior studies also confirm the efficacy of TF in reducing metal artifacts without degrading image quality or increasing the dose [18]. These benefits are particularly significant in postoperative vascular lesion assessments, even in the presence of stents or stent grafts (Figure 3).

Moreover, our evaluation approach expanded beyond conventional methods by incorporating VS and FWHM measurements for the first time in a study involving a TF. However, it was observed that these metrics were significantly lower when compared to those obtained using ST100kV. This is likely due to the method of measuring the VS by drawing a linear line on the SMA, which is influenced by various factors such as noise and contrast. In our study, it seems competitive that the TF100kV group showed significantly lower VS and FWHM, likely due to increased noise and decreased contrast.

Our study had several limitations that warrant consideration. Firstly, it was conducted as a single-center retrospective study with a relatively small cohort size. Secondly, our study did not evaluate the diagnostic confidence and accuracy of spectral filtration in aortic disease, as we did not perform tests on the same patients for both the control and study groups. Additionally, we did not measure diagnostic accuracy based on factors such as BMI, type of lesion, and presence or absence of lesions. The nature of aortic diseases often necessitates immediate intervention, with many cases proceeding directly to surgery following CT imaging. Conducting both a standard dose protocol and a TF low-dose protocol on the same patient would substantially increase radiation exposure. Hence, we opted to compare the TF protocol with a different control group. Future research should focus on assessing the diagnostic performance of TF protocols. Thirdly, minor differences in image quality between the two protocols, such as a blurring appearance in TF images, may have impacted the blinding of the readers. A follow-up study should assess subjective image quality, possibly by employing different reconstruction kernels tailored to the organs being studied. 

## 5. Conclusions

In conclusion, a dedicated low-dose TACTA protocol utilizing a tin filter can achieve a significant reduction in radiation dose while preserving adequate diagnostic image quality.

## Figures and Tables

**Table 1:** Qualitative image quality evaluation criteria.

Score	Image Noise	Image Contrast, Sharpness	Subjective Image Quality
1	Unacceptable noise	Very poor	Issues affecting diagnostic information
2	Above-average increased noise	Suboptimal	Major issues affecting visualization of major structures but diagnosis still possible
3	Average noise in an acceptable image	Average	Minor issues possibly interfering with diagnostic decision making
4	Less-than average noise	Above Average	Minor issues not interfering with diagnostic decision making
5	Minimum or no image noise	Excellent	Excellent image quality without related issues of concern

**Table 2:** Study participant demographics and radiation dose estimates in both cohorts.

Variables	TF100kV	ST100kV	*p*
No. of participants	45 (50)	45 (50)	1.000
Sex (%)			1.000
Male	27 (60)	27 (60)	
Female	18 (40)	18 (40)	
Mean age, years (range)	69.8 ± 12.4	68.1 ± 16.4	0.583
Body mass index (kg/cm^2^)	24.15 ± 3.72	24.30 ± 4.00	0.857
CT findings			0.001
Aortic aneurysm	11 (24.4)	4 (8.9)	
Acute aortic syndrome	5 (11.1)	5 (11.1)	
Postoperative follow up	18 (40.0)	5 (11.1)	
Atherosclerosis	7 (15.6)	20 (44.4)	
Bleeding	1 (2.2)	7 (15.6)	
etc.	3 (6.7) ^a^	4 (8.9) ^b^	
Radiation dose estimates			
DLP (mGy*cm)	128.25 ± 18.18	662.75 ± 181.29	<0.001
CTDIvol (mGy)	1.83 ± 0.25	9.28 ± 2.17	0.001
ED (mSv)	2.31 ± 0.33	11.93 ± 3.26	<0.001
mAs (mGy)	428.71 ± 61.81	376.20 ± 25.73	<0.001

^a^ includes superior mesenteric artery dissection (*n* = 1), vasculitis (*n* = 1), and enterocolitis (*n* = 1), and ^b^ includes enterocolitis (*n* = 3) and cardiomegaly (*n* = 1). Abbreviation: CTDIvol, volume CT dose index; DLP, dose-length product; ED, effective dose; mAs, effective tube current.

**Table 3:** Qualitative analysis of the two image sets.

Qualitative Analysis		TF100kV	ST100kV	*p* Value	Kappa
Image noise	Reader 1	3.87 ± 0.89	4.33 ± 0.64	0.013	0.535
	Reader 2	3.84 ± 0.93	4.36 ± 0.57	0.002	
	Reader mean	3.86 ± 0.89	4.34 ± 0.58	0.026	
Contrast and vessel sharpness	Reader 1	3.89 ± 0.78	4.36 ± 0.65	0.004	0.785
	Reader 2	4.00 ± 0.80	4.38 ± 0.61	0.067	
	Reader mean	3.93 ± 0.74	4.37 ± 0.57	0.008	
Subjective image quality	Reader 1	4.07 ± 0.78	4.20 ± 0.63	0.374	0.693
	Reader 2	4.02 ± 0.81	4.24 ± 0.65	0.154	
	Reader mean	4.04 ± 0.77	4.22 ± 0.62	0.232	

**Table 4:** Quantitative analysis of the two image sets.

Quantitative Analysis	Location	TF100kV	ST100kV	*p* Value
SNR	Thoracic aorta	18.27 ± 3.89	23.16 ± 8.52	0.001
	Abdominal aorta	15.95 ± 3.29	18.13 ± 5.39	0.023
	Right CIA	17.62 ± 4.93	22.25 ± 8.19	0.002
CNR	Thoracic aorta	15.22 ± 3.68	19.72 ± 7.25	<0.001
	Abdominal aorta	13.25 ± 4.88	15.52 ± 4.88	0.011
	Right CIA	14.37 ± 4.47	18.76 ± 7.02	0.001
FOM (mSv^−1^)	Thoracic aorta	36.70 ± 22.77	13.96 ± 13.18	<0.001
	Abdominal aorta	27.89 ± 15.65	7.94 ± 4.82	<0.001
	Right CIA	34.59 ± 27.02	12.56 ± 11.55	<0.001
VS	Proximal SMA	58.50 ± 10.06	64.23 ± 8.12	0.004
FWHM	Proximal SMA	5.89 ± 1.10	6.59 ± 0.89	0.001

## Data Availability

All data are available through the corresponding authors.
